# Pneumococcal conjugate vaccine dose-ranging studies in humans: A systematic review

**DOI:** 10.1016/j.vaccine.2021.07.033

**Published:** 2021-07-31

**Authors:** R.K. Lucinde, G. Ong’ayo, C. Houlihan, C. Bottomley, D Goldblatt, J.A.G. Scott, K.E. Gallagher

**Affiliations:** a KEMRI-Wellcome Trust Research Programme (KWTRP), Centre for Geographic Medical Research - Coast (CGMRC), Kilifi, Kenya; b Division of Infection and Immunity, University College London, London, UK; c Faculty of Epidemiology and Population Health, London School of Hygiene and Tropical Medicine, UK; d Great Ormond Street Institute of Child Health, University College London, UK

**Keywords:** Pneumococcal conjugate vaccines, Dose-range, Systematic review, Immunogenicity

## Abstract

**Background:**

*Streptococcus pneumoniae* is one of the most common bacterial pathogens of infants and young children. Antibody responses against the pneumococcal polysaccharide capsule are the basis of vaccine-mediated protection. We examined the relationship between the dose of polysaccharide in pneumococcal conjugate vaccines (PCVs) and immunogenicity.

**Methods:**

A systematic search of English publications that evaluated the immunogenicity of varying doses of pneumococcal conjugate vaccines was performed in Medline and Embase (Ovid Sp) databases in August 2019. We included only articles that involved administration of pneumococcal conjugate vaccine in humans and assessed the immunogenicity of more than one serotype-specific saccharide dose. Results were synthesised descriptively due to the heterogeneity of product valency, product content and vaccine schedule.

**Results:**

We identified 1691 articles after de-duplication; 9 studies met our inclusion criteria; 2 in adults, 6 in children and 1 in both. Doses of polysaccharide evaluated ranged from 0.44 mcg to 17.6 mcg. In infants, all doses tested elicited IgG geometric mean concentrations (GMCs) above the established correlate of protection (COP; 0.35 mcg/ml). A month after completion of the administered vaccine schedule, 95% confidence intervals of only three out of all the doses evaluated had GMCs that crossed below the COP. In the adult studies, all adults achieved GMCs that would be considered protective in children who have received 3 standard vaccine doses.

**Conclusion:**

For some products, the mean antibody concentrations induced against some pneumococcal serotypes increased with increasing doses of the polysaccharide conjugate, but for other serotypes, there were no clear dose–response relationships or the dose response curves were negative. Fractional doses of polysaccharide which contain less than is included in currently distributed formulations may be useful in the development of higher valency vaccines, or dose-sparing delivery for paediatric use.

## 1 Background

The polysaccharide capsule of *Streptococcus pneumoniae* is the principal target of the mature human response to pneumococcal infection and the reason initial vaccine development focused on pneumococcal polysaccharide vaccines [1]. However, polysaccharides are poor immunogens, especially in infants and the elderly [1,2]. Conjugation of serotype-specific capsule polysaccharides to a carrier protein improves immunogenicity by stimulating T-cell dependent responses [3].

Early conjugate vaccine candidates differed in the dose of saccharide conjugated to the carrier protein, the saccharide chain length, the carrier protein used, the ratio of carrier protein to saccharide, the conjugation method, the adjuvant used and the vaccination schedule [4–27] (Table 1).

Some of the evidence that led to the vaccine formulations in use today has been summarised previously [3]. In brief, polysaccharides were found to be more immunogenic than oligosaccharides [2,28]. Proteins used in other conjugate vaccines, like Tetanus Toxoid (TT) or *Neisseria meningitidis* outer membrane protein (OMPC) generated lower immune responses when used in PCVs compared to other carrier proteins such as Diphtheria Toxoid (DT) and Diphtheria Toxoid mutant (Dip. CRM197) [3]. PCVs using TT, OMPC or protein D seemed to elicit a peaked response (immunogenicity increased with dose until a threshold and then decreased thereafter) or no dose-dependent response. However, candidates using DT or Dip. CRM197 as the carrier protein elicited a linear dose–response relationship and were less likely to induce epitopic B-cell suppression (CIES) than vaccines using TT or OMPC carrier proteins. Additionally, higher valency PCVs using Dip. CRM197 benefit from coadministration with other infant vaccines using DT protein [3].

The need to keep the total saccharide and carrier protein doses low to avoid interference and/or hypo responsiveness, while incorporating multiple serotypes into the vaccine, led to the development of candidates with lower saccharide doses and lower carrier protein load than the Hib conjugate vaccines previously developed [3]. Doses of saccharide in current conjugate vaccines were determined before the correlate of protection was known. Immunogenicity was measured in fold-rises of IgG titres compared to baseline. Relatively low concentrations of serotype-specific IgG (0.35 mcg/ml) in response to vaccine have since been shown to correlate with protection against invasive pneumococcal disease in infants [29], while protection against acquisition of carriage of pneumococci in the nasopharynx may require higher concentrations (2–5 mcg/ml) [30].

As of March 2019, 75% of countries globally had introduced PCV. Since 2010, Gavi, the Vaccine Alliance, has supported PCV introduction in 60 low and middle-income countries (LMICs) [31]. PCV alone represents the largest proportion of the Gavi budget when compared to all other vaccines [32] and, at approximately US$10 per fully immunized child, the most expensive vaccine in the routine vaccination schedule for many LMICs [33]. One approach to reducing the financial cost of PCV programmes is to use a fractional dose at each vaccination but this is only possible if lower doses are sufficiently immunogenic to indicate strong protection. We examined previous literature on the relationship between the dose of polysaccharide in pneumococcal conjugate vaccines (PCVs) and immunogenicity in a systematic review.

## 2 Methods

### 2.1 Search strategy

**Fig. 1:**
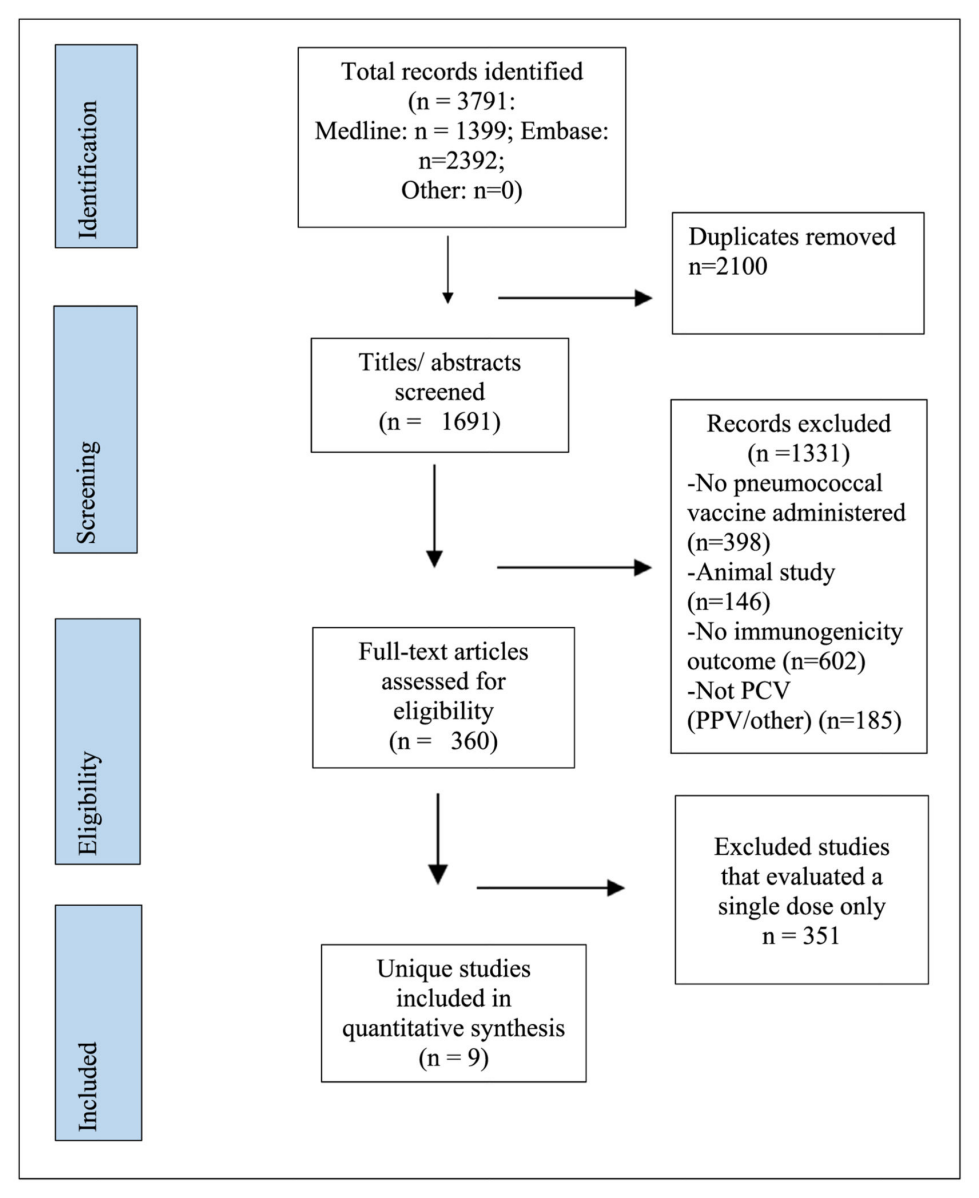
PRISMA flow diagram. This diagram describes the literature search process and inclusion/exclusion criteria used to identify the studies included in this review.

The Preferred Reporting Items for Systematic reviews and Meta-Analyses (PRISMA) guidelines were followed [34]. Medline and Embase databases (Ovid SP) were searched in April 2018 and the search was updated in August 2019. Search terms were built around (1) pneumococcal vaccination/immunisation (2) immunogenicity (3) dose/dosage/dose–response/dose-ranging. The search had no restrictions based on publication date. We included only English-language publications that involved administration of pneumococcal conjugate vaccine in humans and assessed the immunogenicity of more than one serotype-specific saccharide dose (Fig. 1, Supplementary Table 1).

### 2.2 Screening of articles

All articles retrieved from the two databases were exported into Endnote X8 (Clarivate Analytics, PA, USA) and duplicates were automatically and manually removed.

The title and/or abstracts were screened by two reviewers (RKL and KEG) independently (Fig. 1). Full texts were screened by two of three reviewers (KEG, CH and RKL). Articles were excluded if they did not assess >1 dose of polysaccharide conjugate and/or did not report serum IgG concentrations.

### 2.3 Data extraction & synthesis

Data from included articles were extracted into a template in Microsoft Excel 2013. Data on the study population, setting, vaccine formulation, comparison arms/cohorts, schedule, outcome measure(s) and timepoint of outcome measurement were noted alongside any analyses. The qualities of the included studies were evaluated using the Cochrane GRADE system [35].

The studies were not combined in a meta-analysis because of the heterogeneity in the vaccine valency, carrier protein, adjuvant, adjuvant dose, manufacturer and conjugation methods, the vaccination schedule and the population of analysis (children, adults with or without prior vaccination). Instead, serotype-specific dose response curves were estimated using data from studies with the same vaccination schedule and immunogenicity endpoints.

We requested the corresponding authors to provide access to the raw data. Where data was not provided, the proportion of infants and adults with IgG GMCs below the established correlate of protection (0.35 mcg/mL [36]) was estimated from the reported estimates of the geometric mean concentrations to each dose and log-scale standard deviation by assuming a normal distribution. To evaluate whether the assumption of normality was reasonable, the estimated proportions for one of the included studies which provided raw data, Rupp *et* al.’s formulation B, were compared with the reported proportions. The estimated proportions were found to be similar to those reported. Since Rupp *et* al. reported the proportion of responders (rather than proportion non-responders), the proportion of non-responders for their study was calculated as 1-proprotion responders.

## 3 Results

The search identified 3791 articles; 1691 remained after deduplication (Fig. 1). A total of 360 full texts were reviewed; 9 studies were included in the review [2,28,37–43] (Table 2). Of the nine, two studies involved adult populations [41,43], six involved paediatric populations [2,28,38–40,42] and one involved adult and paediatric populations [37].

### 3.1 Quality of included studies

All the included studies were individually randomised controlled trials. The included studies were graded to have high to moderate quality of evidence (Supplementary Table 2). The blinding procedures for four of the nine studies [38–40,42] were not reported. Only five of the nine included studies, [37–39,41,43], mentioned the number of participants withdrawn or lost to follow up prior to the primary endpoint.

### 3.2 Immunogenicity in adult studies

Three studies involved adult populations [37,41,43] (Table 2). Lode *et* al. and Jackson *et* al. studied the immunogenicity of PCV7 (Prevnar ®, Wyeth Vaccines, NY) in healthy adults > 70 years old with no history of PPV [43] and in adults 70–79 years old with a previous history of PPV exposure [41,44] respectively. The vaccines were administered as a single dose with polysaccharide doses ranging from 0.44 to 8.8 mcg for serotypes 4, 9V, 14, 18C, 19F, 23F and 0.88 to 17.6 mcg for serotype 6B. Rupp *et* al. evaluated the safety and immunogenicity of two formulations of PCV15 (Merck Sharp & Dohme Corp) in healthy adults aged 18 to 49 years with no history of either PPV or PCV exposure. The vaccines were administered as a single dose in each group at polysaccharide doses of 2 and 4 mcg. All PCV7 doses evaluated by Lode *et* al. and Jackson *et* al. were also evaluated by Rupp *et* al.

A dose dependent increase in serum IgG GMCs which then plateaued was apparent for serotype 4 for all three adult studies [37,41,43], serotype 6B for two out of three studies [41,43] and for serotype 23F in one of the three studies [41]. The overall IgG GMCs reported for Jackson *et* al. were lower than those reported for Lode *et* al. for all serotypes. IgG GMCs for serotype 9 and 23F reduced at higher doses in Lode *et* al. [43] and for both formulations in Rupp *et* al. [37], while those for serotype 19F, 18C, 9V and 23F for Lode *et* al. [43] increased with higher doses (Supplementary Fig. 1).

Estimated proportions of adults with IgG GMCs below the infant correlate of protection were calculated for the studies which reported IgG GMCs and the confidence intervals around these means, assuming a normal distribution (Supplementary Fig. 2). These proportions ranged between 0.1% (95% confidence interval (CI): 0–17.0%) (Lode *et* al., serotype 18C, dose: 4.4 mcg/mL) and 22.3% (95 %CI: 12.4–36.8%) (Jackson *et* al., serotype 4, dose: 0.44 mcg/mL).

### 3.3 Immunogenicity in paediatric studies

A total of 7 studies involved paediatric populations ranging from 2 to 30 months of age [2,28,37–40,42] (Table 2). Daum *et* al. [28], Ahman *et* al. (1998 and 1999) [39,40] and Zangwill *et* al. [42] evaluated varying doses of experimental PCVs in 3-dose schedules at 2 ,4 and 6 months of age. Steinhoff *et* al. [2] evaluated the immunogenicity of varying doses of PCV2 after a single dose administered at 18–30 months. Anderson *et* al. [38] evaluated varying doses of an experimental PCV3 with two different carrier proteins (Dip. CRM197 and Tetanus Toxoid) after two doses administered at 24 and 26 months. Rupp *et* al. [37] evaluated varying doses of two PCV15 formulations (Merck Sharp & Dohme Corp) after administration of a 4-dose schedule at 2, 4, 6 and 12–15 months of age. Concomitant vaccinations as per national vaccination schedules were allowed for all the studies. As immunogenicity varies with age, the two studies in toddlers [2,38] were not included in the descriptive synthesis as toddlers are not the target population for current routine immunization programmes. The common serotype evaluated by the toddler studies [2,38] was 23F. The proportion of toddlers with > 4-fold increase in IgG GMCs from baseline after a single dose for serotype 23F in these two studies ranged between 20% (group that received 5.1 mcg of PCV)[38] and 94% (group that received 2 mcg of PCV)[2].

**Fig. 2:**
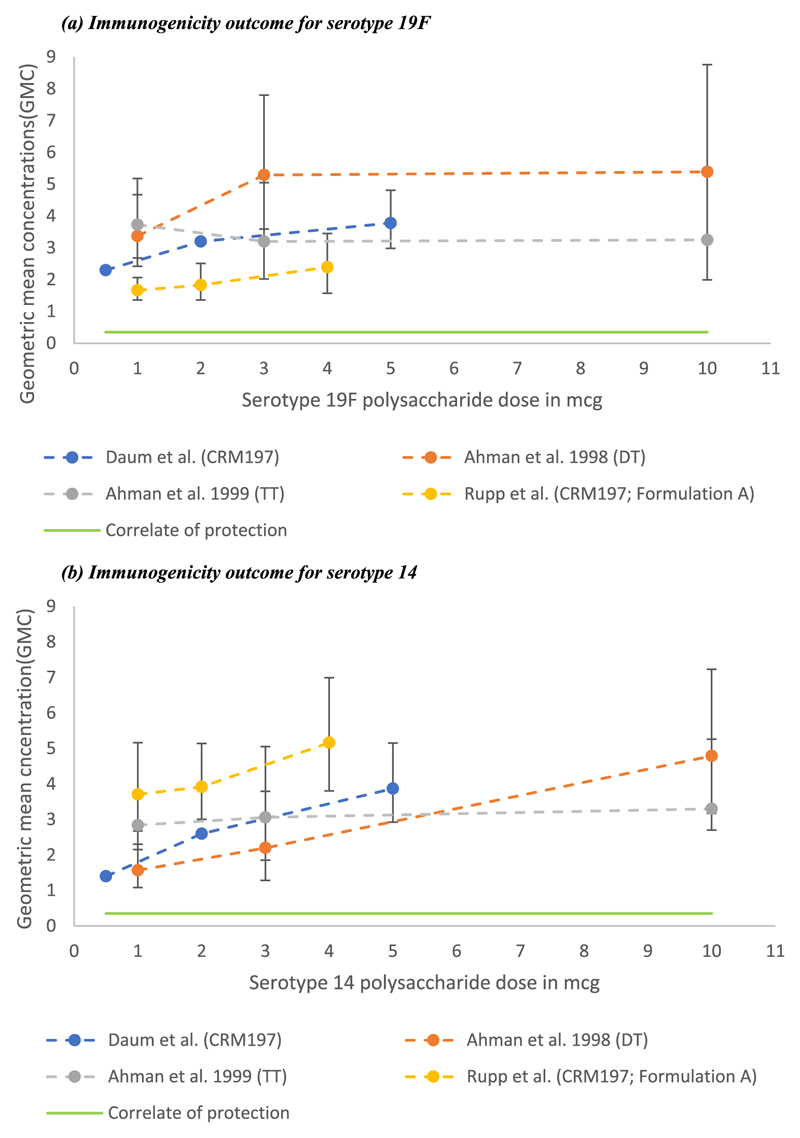

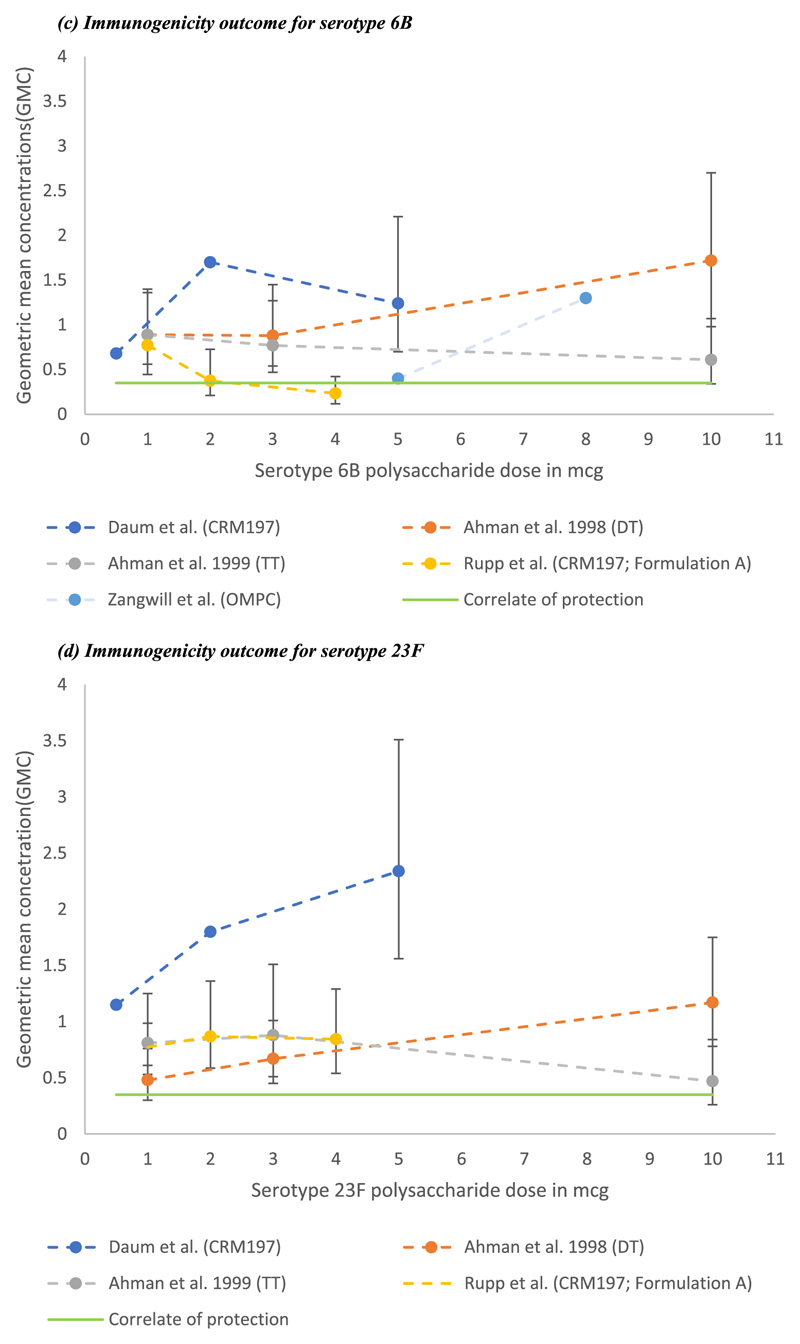
Immunogenicity outcome in paediatric studies. These figures illustrate the various immunogenicity outcomes for some of the included paediatric studies. The round dots represent point estimates i.e. the IgG GMCs reported for each polysaccharide dose evaluated. The limits plotted about the point estimates are margins of error calculated from the point estimates and their 95% confidence intervals. Note: the scale of the axes for 6B and 23F differ from the scale for 19F and 14 due to the difference in range of GMCs. Legend: Publication (vaccine carrier protein) (a) Immunogenicity outcome for serotype 19F. (b) Immunogenicity outcome for serotype 14. (c) Immunogenicity outcome for serotype 6B. (d) Immunogenicity outcome for serotype 23F.

Serotype specific IgG GMCs post final dose in comparable infant populations were plotted against each other for the common serotypes 6B, 14, 19F and 23F using data from Daum *et* al.[28], Ahman *et* al. (1998 and 1999) [39,40] and Rupp *et* al.’s formulation A with 250 mcg of aluminium phosphate([37]. Zangwill *et* al.’s [42] IgG GMCs were included for serotype 6B (Fig. 2). A dose–response effect was apparent for STs 14, 19F and 23F for the Daum *et* al. [28] and Ahman (1998) *et* al. [39] studies.

Confidence intervals around the IgG GMC for serotype 6B and 23F’s highest dose in the Ahman (1999) *et* al. [40] study crossed the correlate of protection as well as those for Ahman (1998) *et* al.’s [39] lowest dose for serotype 23F (Fig. 2).

**Fig. 3:**
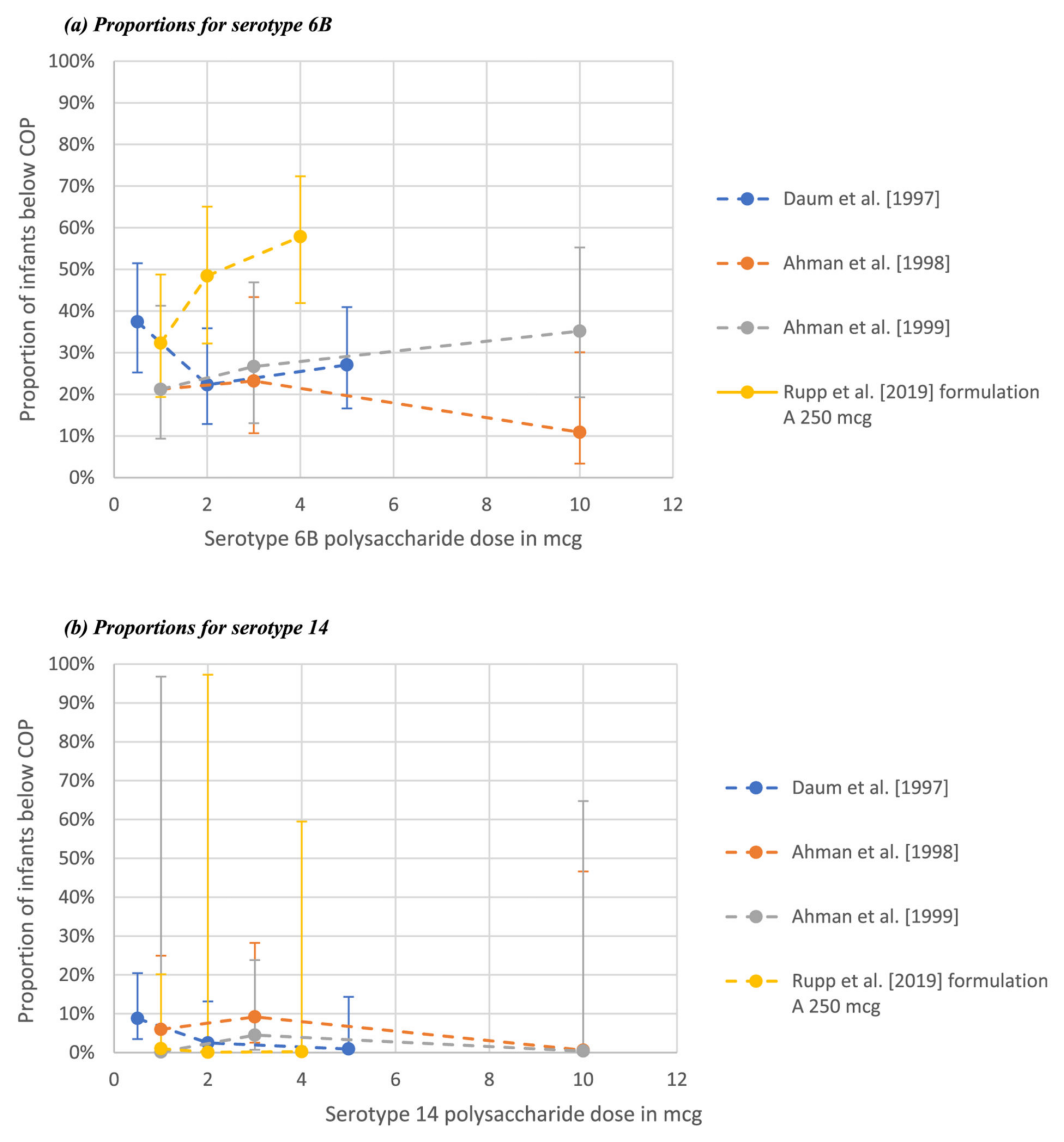

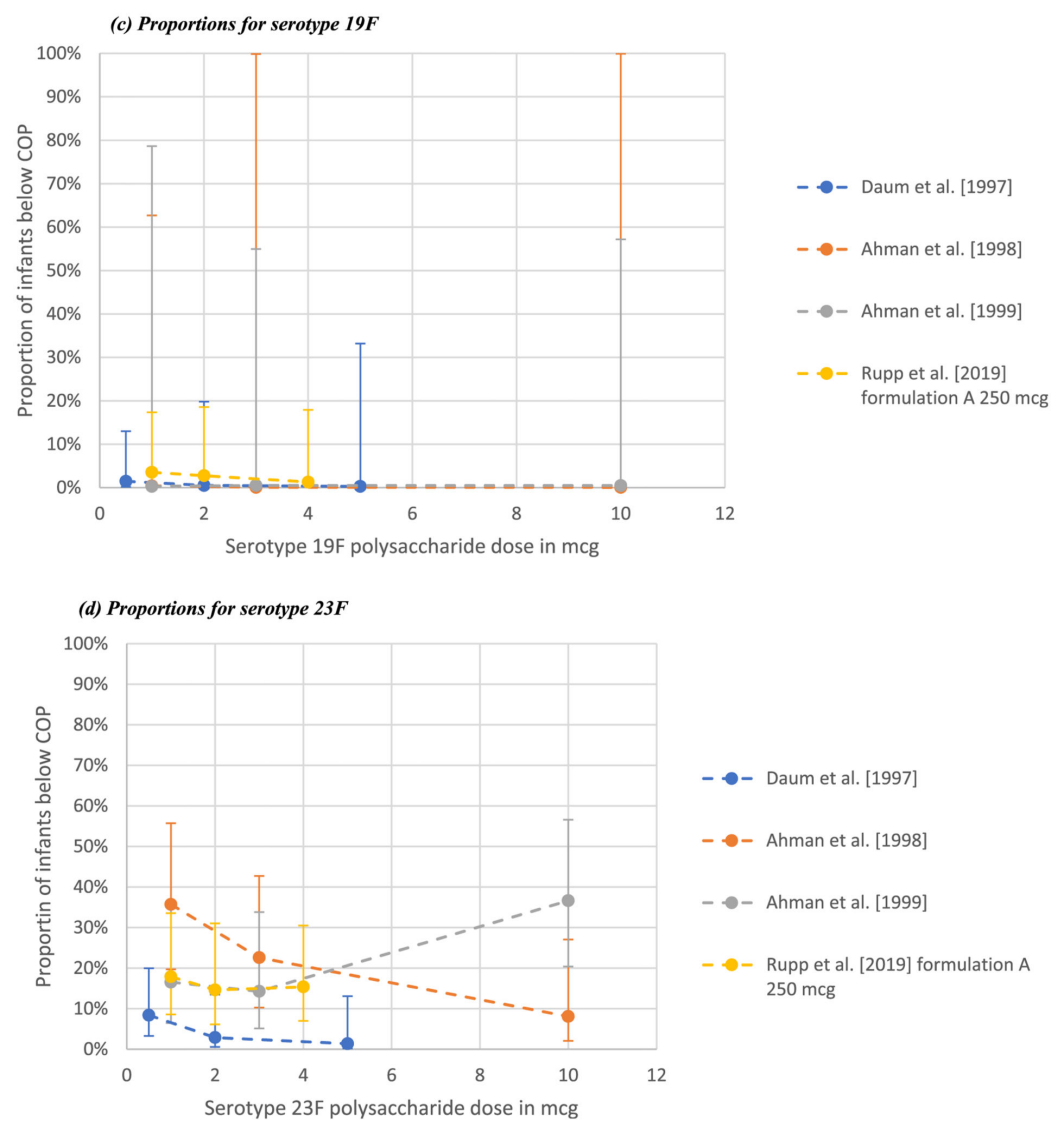
Estimated proportion of infants below correlate of protection (COP). These figures illustrate the proportion of infants below the established COP as estimated from the data extracted. The round dots represent point estimates i.e. The estimated proportion below COP. The limits plotted about the point estimates are margins of error obtained from the difference between the 95% confidence intervals and the respective point estimates on either side. Legend: Publication (vaccine carrier protein) (a) Proportions for serotype 6B. (b) Proportions for serotype 14. (c) Proportions for serotype 19F. (d) Proportions for serotype 23F.

Estimated proportions of infants with IgG GMCs below the correlate of protection (0.35mcg/mL) were calculated for comparable infant studies which reported IgG GMCs and the confidence intervals around these means. Serotype 6B had the highest proportion of infants below the correlate of protection compared to other serotypes (Fig. 3). Increasing doses for STs 6B, 14 and 23F seemed to correspond to a decrease in the proportion of infants below the correlate of protection in the Ahman (1998) et al. trial [39].

### 3.4 Follow-up post primary endpoint in children

The longest follow up reported was 36 months after enrolment [39,40]. A booster dose was administered to children in three studies. All booster doses elicited a strong memory response. Two studies reported that after a polysaccharide vaccine booster, antibody responses post-boost were higher in those who received the lowest vaccine dose in infancy (Table 3).

## 4 Discussion

This review aimed to collate evidence on the immunogenicity of varying doses of serotype specific polysaccharide within PCVs. Nine studies were included after a literature search that was limited to studies in humans that reported immunogenicity outcomes for varying doses. It is likely that more information on dose–response exists but lies unpublished by vaccine manufacturers as part of their research and development data. The studies included were all RCTs and graded to be of moderate to high quality evidence. Some of the studies had small sample sizes per trial arm but the effect of this on the statistical power of the results could not be calculated due to limitations in the data reported e.g. no information on loss to follow up and the IgG GMC variance. The included studies were published between 1994 and 2018. Most studies were published before there was an established immune correlate of protection in children, to inform the study results. The most recent study was of a PCV15 [37] which is currently undergoing adult and paediatric clinical development.

Of the seven paediatric studies included, five administered the study vaccine in a schedule of 3 primary doses (3p + 0) or a schedule of 3 primary doses plus a booster (3p + 1) to infants, starting at 2 months of age i.e. findings may be relevant to current routine infant immunisation schedules. The PCV doses tested ranged between 0.5 and 10 mcg. Only two of these five paediatric studies showed a dose–response where higher ST-specific doses correlated with higher GMCs after the prime vaccinations [37,39]. Paradoxically a clear dose response was not seen for ST6B; however, this serotype is consistently included at higher doses in licensed products than other serotypes, the data supporting this is decision is unclear from the available literature.

When the proportion of children with antibody titres above the established correlate of protection was estimated from the reported GMCs, the confidence intervals around the estimates are wide. Only one of the five studies showed a consistent favourable trend with dose, where the proportion of infants below the correlate of protection (i.e. “unprotected”) decreased with higher doses [39]. The limitations of this approach are acknowledged, the assumption of a normal distribution could be incorrect, despite it being supported by the data visually. Assuming alternative distributions could result in greater or lesser proportions above the correlate of protection. The performance of the assays used by the older studies [2,28,39,40] were not standardised. Because of this, it is unclear how their antibody results relate to the 0.35 mcg/ml threshold and they may not be accurate at the lower limits. Additionally, the established correlate of protection is thought to overestimate the IgG concentrations needed to protect against invasive pneumococcal disease (IPD) caused by serotypes 6A, 6B, 18C and 23F and underestimate the concentration needed to protect against IPD caused by serotypes 1, 3, 7F, 19A and 19F [45,46]. Future PCVs may benefit from being evaluated against ST-specific thresholds rather than a common correlate of protection. However, this review provides some evidence that smaller doses than those included in currently distributed PCVs are immunogenic and could be protective in children.

In all three adult studies, there was a dose response where the highest dose induced the highest immune response [43]. History of pneumococcal polysaccharide vaccine prior to PCV administration could have contributed to the consistently lower IgG GMCs (hyporesponsiveness) in otherwise comparable participants enrolled in the Jackson *et* al. study, compared to the Lode *et* al. study [1,43]. There is no established correlate of protection for adult populations and therefore the clinical implications of the observed dose–response are unclear.

Lower priming doses were reported to give a higher GMCs post-boost, regardless of the vaccination schedule, in two paediatric and two adult studies that assessed this [37,39,40,42]. There are some data from studies of other vaccines that indicate smaller prime doses may elicit better memory responses to a booster dose [48,49]. Although the mechanisms for this are unclear, it is a reminder that measures of immunogenicity one month after the final dose in the series should not be seen in isolation and future studies should assess the impact of dose on immune memory.

This review is limited by the fact that the observed relationships between dose and immunogenicity are heterogenous and much of this variation may be attributable to factors other than the saccharide dose e.g. the carrier protein, the ratio of polysaccharide to carrier protein, the method of conjugation and the adjuvant of choice [3]. The two Ahman *et* al. studies provide a comparison of two carrier protein conjugates across three saccharide doses. In these studies, the TT conjugates [40] show a varied pattern, whereas the DT conjugates showed a dose–response relationship for some STs [39]. Other important factors are the conjugation technique and dose of adjuvant. For example, the Rupp *et* al. studies evaluated varying doses of PCV15 in two formulations that differed in their conjugation method and amount of aluminium hydroxide. One formulation performed better than the other across all serotypes in adults and infants and was selected for further clinical investigation [37]. Interaction with concurrently administered vaccines can also influence immune responses [47]. Despite reporting a satisfactory immune response to a primary series with OMPC as a carrier protein, Zangwill *et* al. [42], reported a negative effect of concurrent immunization with a homologous carrier protein (Hib conjugate vaccine) on the immune response to PCV. In addition to these factors, development of higher valency PCVs will also need to consider the total polysaccharide and carrier protein content to avoid hypo-responsiveness and immune interference e.g., PCV13 has been shown to induce a lower individual immune response compared to PCV7 and this may be due to the increase in total polysaccharide and carrier protein content [3,47].

## 5 Conclusion

In conclusion, for some products, the mean antibody concentrations induced against some pneumococcal serotypes increased with increasing doses of the polysaccharide conjugate, but for other serotypes and other products there was no clear dose–response relationship or the dose response curves were negative. Overall, in children, evidence suggests smaller doses of polysaccharide than those in currently distributed formulations are immunogenic and may be protective. However, the carrier protein content, conjugation technique and adjuvant also determine the quality and quantity of the immune response.

Since development of higher valency PCVs relies on optimization of the polysaccharide dose while minimizing the total polysaccharide and carrier protein content and adjuvant volume [3], evidence of the immunogenicity of these small doses of polysaccharide may be useful in the development of higher valency vaccines, or dose-sparing delivery.

## Figures and Tables

**Table 1:** Candidate Pneumococcal Conjugate Vaccine formulation (pre- and post-licensure).

Licensure status	Licensed as PCV7							Licensed in PCV10			Licensed in PCV13			Not licensed		Conjugate protein	Adjuvant
Pneumococcal serotype saccharide, dose (μg)	4	6B	9V	14	18C	19F	23F	1	5	7F	3	6A	19A	22F	33F		
Pre-licensure vaccine candidates [Manufacturer, year of earliest appearance in publication]																	
PCV4 [4] [Merck, 1995]		1		1		1	1									Mening. (B) OMPC	Aluminium hydroxide
PCV7 [5,6] [Merck, 1995]	1	2.5	1	1	1	1	1									Mening. (B) OMPC	Aluminium hydroxide
PCV7 [7,8] [Merck, 1996]	1	3.5	1.5–2	1	1	2–2.5	1									Mening. (B) OMPC	Aluminium hydroxide
PCV5 [9–12] [Lederle, 1996]		10		10	10	10	10									Dip. CRM197	Aluminium hydroxide
PCV5 [13] [Lederle, 1996]		5		5	5	5	5									Dip. CRM197	Aluminium phosphate
PCV4 [14] [Pasteur Merieux, 1997]		3		3		3	3									TT or Dip. Toxoid	
PCV7 [15,16] [Wyeth, 1998]	2	4	2	2	2	2	2									Dip. CRM197	Aluminium phosphate
PCV9 [17–19] [Wyeth-Lederle, 1999]	2	4	2	2	2	2	2	2	2							Dip. CRM197	Aluminium phosphate
PCV11 [20–22] [Aventis Pasteur, 2001]	1	10	1	3	3	1	1	1	1	1	3					TT (ST 1, 4, 5, 7F, 9V, 19F, 23F), Dip. Toxoid (ST 3, 6B, 14, 18C)	Aluminium hydroxide
PCV8 [23] [Aventis Pasteur, 2004]	3	3	3	3	3	3	3				3					Dip. Toxoid	
PCV8 [23] [Aventis Pasteur, 2004]	1	1	1	1	1	1	1				1					TT	
PCV11 [24] [GSK, 2008]	1	1	1	1	1	1	1	1	1	1	1					D (NTHib)	
PCV7 [25] [ Centre for Bimolecular Chemistry Cuba, 2014]		4		2	2	2	2	2	2							TT	Aluminium phosphate
PCV15 [26,27] [Merck Sharpe & Dohme, 2015]	2	4	2	2	2	2	2	2	2	2	2	2	2	2	2	Dip. CRM197	Aluminium phosphate
Licensed products [year of licensure]:																	
PCV7 (Pfizer/Wyeth; 2000)	2.2	4.4	2.2	2.2	2.2	2.2	2.2									Dip. CRM197	Aluminium phosphate
PCV10 (GSK, 2009)	3.0	1.0	1.0	1.0	3.0	3.0	1.0	1.0	1.0	1.0						D (NTHib), Dip, TT	Aluminium phosphate
PCV13 (Pfizer/Wyeth; 2010)	2.2	4.4	2.2	2.2	2.2	2.2	2.2	2.2	2.2	2.2	2.2	2.2	2.2			Dip. CRM197	Aluminium phosphate

Abbreviations: CRM197: non-toxic mutant of Diphtheria toxin; D(NTHib): Protein D of non-typeable Haemophilus influenzae type b; DT: Diphtheria Toxin; OMPC: outer membrane protein complex of *Neisseria meningitidis* serotype B; TT: Tetanus Toxin.

1 PCV10 (GSK) product.

**Table 2:** Summary of included studies.

Reference	Population (age at enrolment)	Vaccine schedule	Total Sample Size	Arms	PCV valency (targeted serotypes)	Manufacturing company	Carrier protein	Adjuvant	Doses tested (mcg)^1^	Timepoint of primary outcome
Steinhoff (1994) [2]	American children (18 – 30 months)	Single dose	118	7	PCV 2 (6B, 23F)	Lederle	DT	Aluminium Phosphate	2, 10	1-month post dose
Daum (1997) [28]	American infants (2–3 months)	2, 4, 6 months	400	7	PCV 5 (6B, 14, 18C, 19F, 23F)	Wyeth-Lederle	DT	Aluminium Phosphate	0.5, 2, 5	1-month post dose 3
Ahman (1998) [39]	Finnish infants (9–13 weeks)	2, 4, 6 months	125	4	PCV 4 (6B, 14, 19F, 23F)	Pasteur Merieux	DT	Not stated	1, 3, 10	1-month post dose 3
Ahman (1999) [40]	Finnish infants (9-13 weeks)	2, 4, 6 months	75	3	PCV 4 (6B, 14, 19F, 23F)	Pasteur Merieux	TT	Not stated	1, 3, 10	1-month post dose 3
Zangwill (2003) [42]	American infants (2 months)	2, 4, 6, 12 months	240	3	PCV 7 (4, 6B, 9V, 14, 18C, 19F, 23F)	Merck &Co	OMPC (123 vs 110 mcg)	Aluminium Phosphate	6B: 5, 8 23F: 4 18C, 19F: 2 4, 9V, 14: 1	1-month post dose 3
Anderson (2003) [38]	American children (2 years)	24, 26 months	112	5	PCV 3 (6A, 14, 19F)	Eli Lilly &Co	CRM197	None	6A: 6.7, 15.8 14: 5.3, 12.7 19F: 5, 12.5	1-month post dose 2
Rupp (2019) [37]	American infants (6–12 weeks)	2, 4, 6, 12-15 months	404	8	PCV 15 Formulation A^2^ (1, 3, 4, 5, 6A, 6B, 7F, 9V, 14, 18C, 19A, 19F, 22F, 23F, 33F)	Merck & Co	CRM197	Aluminium Phosphate (125 vs 250 mcg)	Ĵ, 2, 4 6B: 2, 4, 8	1-month post dose 3
	American infants (6 – 12 weeks)	2, 4, 6, 12-15 months			PCV 15 Formulation B^2^ (1, 3, 4, 5, 6A, 6B, 7F, 9V, 14, 18C, 19A, 19F, 22F, 23F, 33F)	Merck & Co	CRM197	Aluminium Phosphate (125 vs 250 mcg)	2, 4 6B: 4, 8	1-month post dose 3
	American adults (18–49 years) with no history of PPV or PCV	Single dose	80	4	PCV 15 Formulation A^2^ (1, 3, 4, 5, 6A, 6B, 7F, 9V, 14, 18C, 19A, 19F, 22F, 23F, 33F)	Merck & Co	CRM197	Aluminium Phosphate (125 vs 250 mcg)	2, 4	1-month post dose
	American adults (18–49 years) with no history of PPV or PCV	Single dose			PCV 15 Formulation B^2^ (1, 3, 4, 5, 6A, 6B, 7F, 9V, 14, 18C, 19A, 19F, 22F, 23F, 33F)	Merck & Co	CRM197	Aluminium Phosphate (125 vs 250 mcg)	2, 4	1-month post dose
Lode (2011) [43]	German adults (>70 years) with no history of PPV or PCV‘	Single dose	443	4	PCV 7 (4, 6B, 9V, 14, 18C, 19F, 23F)	Wyeth Vaccines	CRM197	Aluminium Phosphate (125 vs 250 mcg)	0.44, 2.2, 4.4, 8.8 6B: 0.88, 4.4, 8.8, 17.6	1-month post dose
Jackson (2007) [41,44]	Adults (70–79 years) with history of PPV at least 5 years prior	Single dose	220	5	PCV 7 (4, 6B, 9V, 14, 18C, 19F, 23F)	Wyeth Vaccines	CRM197	Aluminium Phosphate (125 vs 250 mcg)	0.44, 2.2, 4.4, 8.8 6B: 0.88, 4.4, 8.8, 17.6	1-month post dose

Abbreviations: CRM 197: non-toxic mutant of Diphtheria toxin; DT: Diphtheria Toxin; OMPC: outer membrane protein complex of *Neisseria meningitidis* serotype B; TT: Tetanus Toxin.

1 Doses stated are for all serotypes unless named serotypes are specified.

2 The two Rupp *et* al. formulations were conjugated differently. However, each formulation evaluated either 125 or 250 mcg aluminium phosphate adjuvant.

**Table 3:** Follow-up post primary series-paediatric studies.

Study	Longest follow-up	Booster dose administered	Antibody levels pre-boost	Response to booster dose
Ahman et al. [39] PCV4 with DT carrier protein	36 months	PncPS at 14 months^1^	At 14 months significant waning of IgG GMCs against STs 6B, 14 and 19F but not against 23F. No significant difference in titres by original dose of PCV.	3–24-fold increase in IgG GMCs. Booster response was highest in those who received the lowest doses in infancy.
Ahman et al. [40] PCV4 with TT carrier protein	36 months	PncPS at 14 months	At 14 months significant waning of IgG GMCs No significant difference in titres by original dose of PCV.	2.15–12-fold increase in IgG GMCs Booster response was highest in those who received the lowest doses in infancy
Zangwill et al. [42] PCV7 with OMPC carrier protein	13 months	PCV at 12 months	Antibody decline was substantial but comparable in all groups	4.3–6.5-fold rise, comparable in all groups

Abbreviations: DT: Diphtheria toxoid; GMC: geometric mean concentration; IgG: immunoglobulin; OMPC: outer membrane protein complex of Neisseria meningitidis serotype B; PCV: pneumococcal conjugate vaccine; PncPS: Pneumococcal Polysaccharide Vaccine; ST: serotype; TT: tetanus toxoid.

1 Boost dose was administered to all infants who received PCV in infancy (not placebo).
